# Oral Swab Testing With Xpert MTB/RIF Ultra for the Diagnosis of Tuberculosis in Children Aged <5 Years in Uganda: An Exploratory Interim Analysis of Diagnostic Accuracy in the NOD-pedFEND Cohort

**DOI:** 10.1093/ofid/ofaf206

**Published:** 2025-04-08

**Authors:** Nisreen Khambati, Kanyange Angel Moureen, Francesca Wanda Basile, Rudie Desravines, Nathan Mudrak, Rebecca Post, Emmanuel Nasinghe, Rutvi Upadhyay, Sandra Ruth Babirye, Stephannie Nabuduwa, Germine Nakayita, Farag Kakyama Luwambya, Malik Koire, Allen Nabisere, John Paul Lubega, Rose Nabirye, Margaretha de Vos, Adam Penn-Nicholson, Emily Douglass, Prossy Mbekeeka, Derek Armstrong, H Simon Schaaf, Megan Palmer, Eric Wobudeya, Soyeon Kim, Morten Ruhwald, David Alland, Susan E Dorman, Jerrold Ellner, Moses Joloba, Adeodata R Kekitiinwa, Else Margreet Bijker, Grace Paul Kisitu, Rinn Song, and the NOD-pedFEND Consortium, Gerald Agaba Muzorah, Gideon Ahimbisibwe, Sharley Aloyo, Sheillah Ansiima, Kiranjot Arora, Henry Balwa, Kisegerwa Bashir, April Borkman, Eric Bugumirwa, George Haumba, David Hom, Florence Kalawa, Florence Kalembe, Samuel Kasibante, Nakitto Aisha Kawwoya, Daisy Kyamulabi, Rose Magala, Frank Matovu, Angella Mirembe, Benedicto Mugabi, Nelson Muhumuza, Levi Mwanga, Mary Nakagwa, Brenda Sharon Nakalanda, Lydia Nakiyingi, Aminah Nakyazze, Rose Namaganda, Hellen Nassolo, Claire Night, Gloria Ninsiima, Israel Odongo, Kamulegeya Rogers, Willy Ssengooba, Jessica Tagobera, Abner Tagoola, Ann Tufariello, Agnes Turyamubona

**Affiliations:** Oxford Vaccine Group, Department of Paediatrics, University of Oxford, Oxford, UK; Department of Immunology and Molecular Biology, College of Health Sciences, Makerere University, Kampala, Uganda; Oxford Vaccine Group, Department of Paediatrics, University of Oxford, Oxford, UK; Frontier Science Foundation, Brookline, Massachusetts, USA; Oxford Vaccine Group, Department of Paediatrics, University of Oxford, Oxford, UK; Frontier Science Foundation, Brookline, Massachusetts, USA; Department of Immunology and Molecular Biology, College of Health Sciences, Makerere University, Kampala, Uganda; Integrated Biorepository of the H3Africa Uganda, Kampala, Uganda; Division of Infectious Diseases, Center for Emerging Pathogens, New Jersey Medical School, Rutgers University, Newark, New Jersey, USA; Department of Immunology and Molecular Biology, College of Health Sciences, Makerere University, Kampala, Uganda; Department of Immunology and Molecular Biology, College of Health Sciences, Makerere University, Kampala, Uganda; Integrated Biorepository of the H3Africa Uganda, Kampala, Uganda; Mycobacteriology (BSL-3) Laboratory, Makerere University Biomedical Research Centre, Kampala, Uganda; Baylor College of Medicine Children's Foundation–Uganda, Kampala, Uganda; Baylor College of Medicine Children's Foundation–Uganda, Kampala, Uganda; Department of Paediatrics and Child Health, Jinja Regional Referral Hospital, Jinja, Uganda; Department of Paediatrics and Child Health, Jinja Regional Referral Hospital, Jinja, Uganda; Department of Paediatrics and Child Health, Jinja Regional Referral Hospital, Jinja, Uganda; FIND, Geneva, Switzerland; FIND, Geneva, Switzerland; Division of Infectious Diseases, Center for Emerging Pathogens, New Jersey Medical School, Rutgers University, Newark, New Jersey, USA; Department of Paediatrics and Child Health, Jinja Regional Referral Hospital, Jinja, Uganda; Department of Pathology, Johns Hopkins School of Medicine, Baltimore, MD, USA; Department of Paediatrics and Child Health, Desmond Tutu TB Centre, Stellenbosch University, Cape Town, South Africa; Department of Paediatrics and Child Health, Desmond Tutu TB Centre, Stellenbosch University, Cape Town, South Africa; Mulago National Referral Hospital, Kampala, Uganda; Frontier Science Foundation, Brookline, Massachusetts, USA; FIND, Geneva, Switzerland; Public Health Research Institute, New Jersey Medical School, Rutgers University, Newark, New Jersey, USA; Medical University of South Carolina, Charleston, South Carolina, USA; Division of Infectious Diseases, Center for Emerging Pathogens, New Jersey Medical School, Rutgers University, Newark, New Jersey, USA; Department of Immunology and Molecular Biology, College of Health Sciences, Makerere University, Kampala, Uganda; Integrated Biorepository of the H3Africa Uganda, Kampala, Uganda; Baylor College of Medicine Children's Foundation–Uganda, Kampala, Uganda; Oxford Vaccine Group, Department of Paediatrics, University of Oxford, Oxford, UK; Department of Paediatrics, Maastricht University Medical Centre, MosaKids Children's Hospital, Maastricht, the Netherlands; Baylor College of Medicine Children's Foundation–Uganda, Kampala, Uganda; Oxford Vaccine Group, Department of Paediatrics, University of Oxford, Oxford, UK

**Keywords:** children, diagnosis, oral swab, tuberculosis, Xpert MTB/RIF Ultra

## Abstract

**Background:**

Obtaining respiratory samples to diagnose tuberculosis in young children is challenging. Oral swabs are alternative noninvasive specimens for microbiology.

**Methods:**

We conducted an interim prospective diagnostic accuracy evaluation of Xpert MTB/RIF Ultra (Ultra) on oral swabs for pulmonary tuberculosis in children aged <5 years in Uganda. Most children had 2 consecutive swabs collected in a single cryovial (double swabs). Reference tests consisted of Ultra and culture on 2 nasopharyngeal aspirates and 1 gastric aspirate and Ultra on 1 stool. Children were classified as having confirmed tuberculosis, unconfirmed tuberculosis, or unlikely tuberculosis per the National Institutes of Health. Diagnostic accuracy was determined against a microbiological reference standard and a composite reference standard.

**Results:**

From August 2021 to February 2024, 444 children were enrolled, of whom 399 had complete classifications: 33 had confirmed tuberculosis, 269 had unconfirmed tuberculosis, 70 had unlikely tuberculosis, and 27 were unclassifiable. The median age was 16 months and 17% had HIV. Most children (398/399) had oral swabs collected, all with conclusive Ultra results. The sensitivity of double swabs was 6.9% with a microbiological reference standard (95% CI, 1.9%–22.0%) and 1.8% with a composite reference standard (95% CI, .8%–4.1%). Specificity was at least 99%. Swabs detected tuberculosis in 4 children with negative reference test results, of whom 3 had unconfirmed tuberculosis.

**Conclusions:**

The low sensitivity of Ultra on double swabs precludes its role as a principal diagnostic approach in young children. However, detection of tuberculosis in children who were not otherwise microbiologically diagnosed suggests the utility of oral swabs as add-on samples to increase yield.

## Teaser

Based on 398 children aged <5 years with presumptive tuberculosis in Uganda, the sensitivity of Xpert MTB/RIF Ultra on 2 consecutive oral swabs collected in a single cryovial for the microbiological diagnosis of pulmonary tuberculosis was 7%.

In 2023, tuberculosis (TB) was the leading cause of death from an infectious disease, with approximately 10.8 million people falling ill from TB and 1.25 million deaths [1]. Pediatric TB accounts for 12% of the global burden [2], although this is likely an underestimate, with diagnosis in children aged <5 years posing unique challenges. Clinical features of pediatric TB overlap with other childhood diseases, and chest radiographic features are often nonspecific and subject to variable interpretation [3, 4]. Microbiological tests such as mycobacterial culture and nucleic acid amplification tests (NAATs) have limitations in young children due to paucibacillary disease and difficulty in respiratory sample collection [5]. This underdiagnosis contributes to substantial mortality among young children [6], underscoring the need for easier-to-collect diagnostic samples. As a high-burden country for TB and HIV-associated TB [7], Uganda faces a significant challenge.

Recent studies have demonstrated that *Mycobacterium tuberculosis* (*Mtb*) DNA on the oral epithelium can be detected by oral swabs in TB disease by NAATs [8–14], with the potential scalability of Xpert MTB/RIF Ultra (Ultra) as a low-complexity NAAT endorsed by the World Health Organization. Although induced sputum, gastric aspirates (GAs), and nasopharyngeal aspirates (NPAs) enable microbiological diagnosis in young children who cannot provide sputum spontaneously, the procedures can be uncomfortable and require training, equipment, and infection prevention measures. In contrast, oral swab collection is noninvasive, simple, and painless and does not generate aerosols. A recent study on 18 health care workers' preferences in South Africa found that tongue swabbing was deemed more acceptable than sputum sampling, especially for children and infants [15].

Despite the promise of Ultra on oral swabs, research gaps remain, including the optimal swab-processing method and a lack of prospective data in children [16]. Protocols with the Cepheid Sample Reagent (SR) to process swabs were originally optimized for testing sputum. Alternative SR-free methods, such as heat inactivation of the swab, may yield higher sensitivity [12], although pediatric data have not been published. Despite the increasing number of adult swab studies, there are only 4 in children [9, 11, 14, 17], with 1 using Ultra [11]. Additionally, while some studies demonstrated that multiple swabs can increase diagnostic yield [9, 12], there are no pediatric data on Ultra on 2 swabs collected simultaneously and combined into 1 tube, which requires only 1 Ultra test and could be more feasible programmatically than collection over different time points or days. Addressing these gaps, this interim analysis evaluates the diagnostic accuracy of Ultra on oral swabs for pulmonary TB using different processing methods in children aged <5 years in Uganda. Our interim analysis provides timely data for a rapidly growing area of research that can inform meta-analyses and policy decisions in young children, where data are scarce.

## METHODS

### Study Design and Population

This diagnostic accuracy study was nested within NOD-pedFEND, a National Institutes of Health (NIH)–funded study evaluating novel diagnostic strategies in children aged <5 years in Uganda. Prospective enrollment in NOD-pedFEND began in August 2021, and follow-ups are ongoing until July 2025, with a projected enrollment of 640. A detailed study protocol is available on request. Enrollment was conducted at the Baylor Uganda Children's Clinical Center of Excellence in Kampala and Nalufenya Children's Hospital in Jinja. Children aged <5 years with presumptive pulmonary TB disease were consecutively enrolled if they presented with any of the following: persistent cough for >2 weeks not responding to antibiotics, persistent and unexplained fever or night sweats for >1 week not responding to antibiotics, and unexplained weight loss or failure to thrive. Children were excluded if they weighed <3 kg, the parent/guardian was unwilling to provide informed consent, study procedures were considered an undue risk to life, they had received treatment for TB disease or latent TB in the previous 6 months, or they were unavailable for follow-up visits. Inpatients and outpatients were recruited. The study protocol and informed consent documents were approved by institutional review boards at all partnering sites (Makerere University, Rutgers University, and University of Oxford).

### Study Procedures and Sample Collection

**Figure 1.**
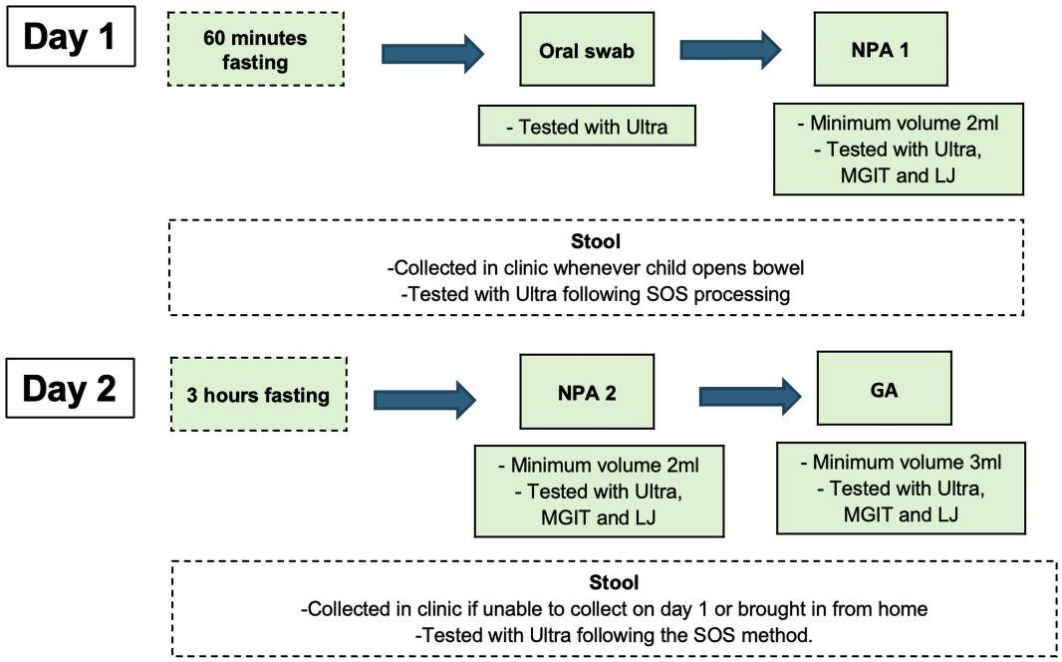
Summary of oral swab and tuberculosis microbiological reference specimen collection. Abbreviations: GA, gastric aspirate; LJ, Löwenstein-Jensen; MGIT, Mycobacterial Growth Indicator Tube 960; NPA, nasopharyngeal aspirate; SOS, Simple One Step; Ultra, Xpert MTB/RIF Ultra.

A standardized clinical, radiologic, and laboratory workup was conducted at baseline, including a medical history, physical examination, TB risk exposure assessment, HIV and malaria testing, and chest radiography. HIV status was determined per national guidelines [18]. The microbiological reference standard (MRS) for TB confirmation was based on a positive Ultra, Mycobacterial Growth Indicator Tube 960, and/or Löwenstein-Jensen culture result on 1 GA and/or 1 of 2 NPA samples collected on consecutive days and/or a positive Ultra result on a stool specimen processed via the Simple One Step method [19]. NPAs were collected after 1 hour of fasting and GA after at least 3 hours of fasting, with each specimen processed by the standard N-acetyl L-cysteine sodium hydroxide method and the subsequent pellet divided for testing with Mycobacterial Growth Indicator Tube 960 and Löwenstein-Jensen culture and Ultra. Stool samples were collected in clinic, or if not possible, parents were given a container to collect at home and bring in the next day. Reference specimens were kept in cold chain during transport to the laboratory (2–8 °C) and tested prospectively per the manufacturers' instructions. Oral swab collection involved swabbing FLOQswabs (COPAN Diagnostics) over the dorsum of the tongue, the inside of both cheeks, and below the tongue, over approximately 15 seconds. Children refrained from eating, drinking, and brushing teeth for at least 1 hour before swab collection, which occurred prior to NPA or GA collection. Figure 1 summarizes the order of oral swab and reference specimen collection.

Children started TB treatment after sample collection according to the attending clinician, who was blinded to swab Ultra results. All children were followed up at 2 weeks, 2 months, and 6 months or the end of TB treatment, whichever was later. Clinical symptoms, examination findings, nutritional status, and treatment adherence were assessed at each follow-up visit. The tuberculin skin test was performed at the week 2 visit and interpreted within 48 to 72 hours [20].

### Oral Swab Laboratory Methods

The number of swabs collected, the timing of Ultra testing, and the processing method changed over time based on emerging laboratory data from unpublished adult studies (Supplementary Table 1). Between August 2021 and December 2021, a single oral swab was collected. Thereafter, 2 swabs were collected successively (one after another) and combined into a single cryovial (double swab). Initially, swabs were immediately frozen at −80 °C and tested later in batches. From August 2022 onward, double swabs underwent prospective testing within 24 hours of collection. When the swab specimen could not be tested immediately, cold chain was maintained (2–8 °C) to prevent degradation of DNA. In terms of processing, from August 2021, swabs were stored and transported in 0.9 mL of phosphate-buffered saline. A total of 1.8 mL of Cepheid SR was added to the swab-containing cryovial (2:1 ratio), and following 2 rounds of vortexing for 10 seconds and incubation at room temperature (first round, 10 minutes; second round, 5 minutes), the total sample mixture (2.7 mL) was pipetted into the Ultra cartridge for testing per the manufacturer's instructions. From July 2023, swabs were instead transported in 0.9 mL of Tris EDTA buffer and processed with the heat inactivation method. This involved boiling the swab-containing cryovial in a heating block for 10 minutes at 95 °C, followed by vortexing for 10 seconds after the sample had cooled and then centrifugation at 15 000 rpm for 30 seconds. Finally, 0.5 mL of swab solution and 1.5 mL of fresh Tris EDTA buffer were pipetted into the Ultra cartridge. Laboratory staff conducting Ultra testing on oral swabs were blinded to clinical information and microbiological reference results.

### Data Analysis

#### NIH Clinical Classifications

Children were classified per the 2015 NIH consensus statements for pediatric diagnostic TB studies [21], described in detail in Supplementary Figure 1. Briefly, pediatric cases were classified as confirmed TB if microbiologically diagnosed on NPA, GA, or stool (ie, excluding oral swabs); unconfirmed TB in the presence of clinical, radiologic, and immunologic features suggestive of TB but negative microbiological testing results; and unlikely TB if criteria for confirmed and unconfirmed TB were not met and at least 1 follow-up visit and chest x-ray (CXR) of sufficient quality were available. Children who did not fall into these categories were deemed unclassifiable. Digital CXRs were retrospectively assessed by an independent and blinded 3-person panel experienced in reviewing pediatric CXRs, with agreement between 2 readers needed to establish the final radiologic status.

#### Diagnostic Accuracy

Sensitivity and specificity were estimated with simple proportions and Wilson score 2-sided confidence interval at the 95% level for all oral swabs and double swabs alone. Three reference standards were derived from the NIH classifications (Supplementary Figure 1). The strict reference standard included children with close-to-definite disease status—namely, confirmed TB as positive and unlikely TB as negative with at least 6 negative microbiological test results. The MRS considered confirmed TB as positive and unconfirmed and unlikely TB as negative with at least 6 negative test results. Finally, the composite reference standard (CRS) defined confirmed and unconfirmed TB as positive and unlikely TB as negative. Subgroup analyses were conducted by HIV infection, malnutrition status, age, and processing method. Per the World Health Organization [22], moderate acute malnutrition was defined as a sex-specific weight-for-height/length *z* score ≤−2 and ≥−3 or a mid upper arm circumference ≥11.5 cm and <12.5 cm if age >6 months. Severe acute malnutrition was defined as a weight-for-height/length *z* score <−3, the presence of bilateral pitting edema, or a mid upper arm circumference <11.5 cm if age >6 months. Only children with a valid Ultra swab result were included in swab diagnostic accuracy estimates. Finally, the diagnostic yield was estimated as the proportion of children identified as having TB among those eligible for testing. Data were analyzed in R software (version 4.3.1) and SAS (version 9.4; SAS Institute).

## RESULTS

**Figure 2.**
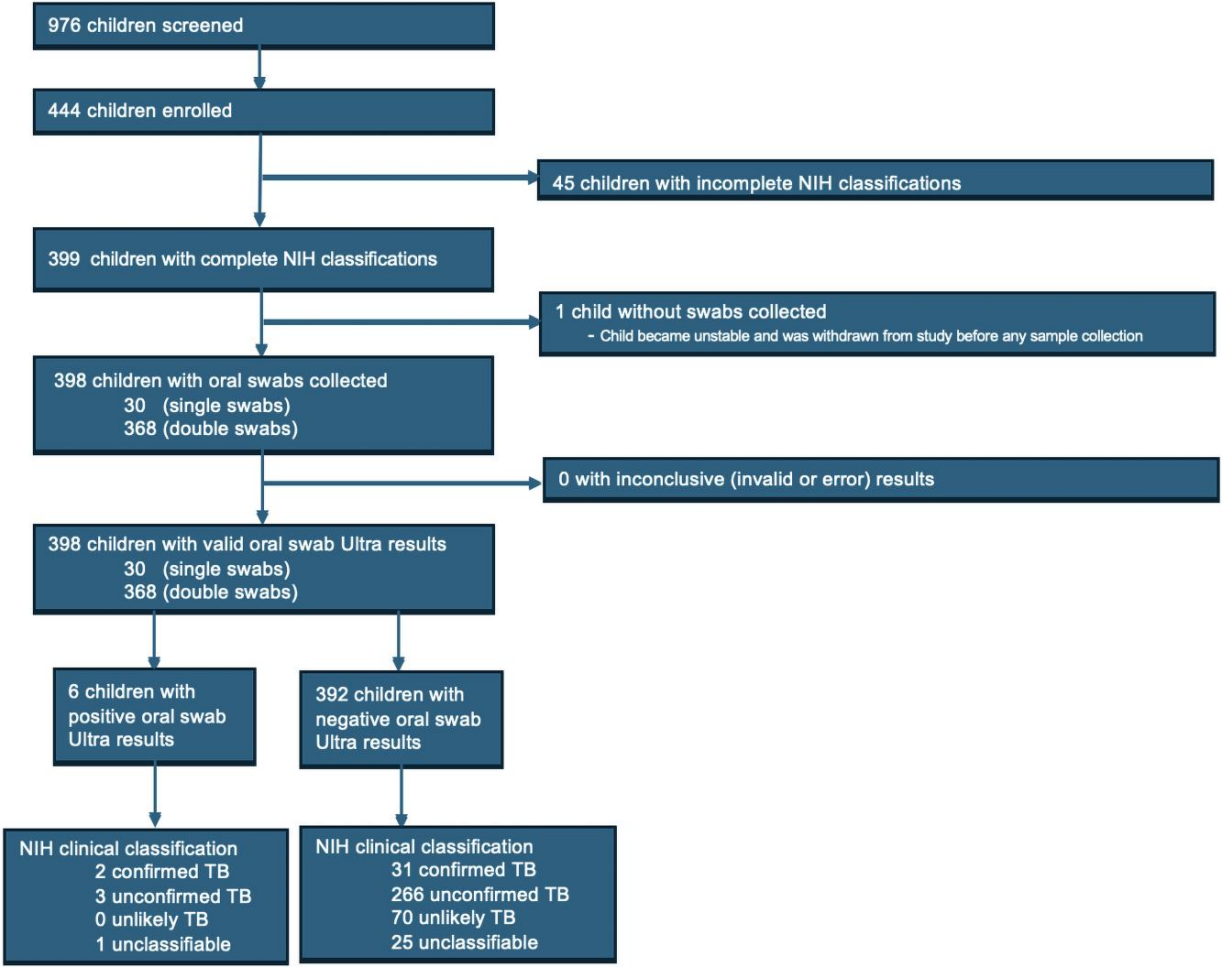
Flow diagram showing the numbers of children enrolled from 17 August 2021 to 29 February 2024 and analyzed. Abbreviations: NIH, National Institutes of Health; TB, tuberculosis.

From 17 August 2021 to 29 February 2024, 444 children were enrolled in NOD-pedFEND from Uganda, of which 399 had complete NIH clinical classifications at the time of writing and were included in the analysis (Figure 2). Of these, 33 (8%) had confirmed pulmonary TB, 269 (67%) had unconfirmed TB, 70 (18%) had unlikely TB, and 27 (7%) were unclassifiable. Among children with confirmed TB, 5 (15%) were Ultra and culture positive, 25 (76%) were positive only with Ultra, and 2 (6%) were culture positive only. Two (6%) children had rifampicin-resistant TB.

The median age across the cohort was 16 months (IQR, 12–35 months), and 48% (191/399) were female. Furthermore, 17% (67/399) were children with HIV (CWH), 47% (188/399) had severe acute malnutrition, 56% (222/399) were hospitalized, and 99% (394/399) reported never having had TB (Table 1). A high proportion of children had started TB treatment (303/399, 76%). In 398 of 399 children, oral swabs were collected successfully. The child without a swab collected was withdrawn before sample collection due to deterioration in clinical status. Most children had double swabs collected (368/398, 92%) and swabs tested prospectively (289/398, 73%), reflecting early protocol changes (Supplementary Table 1). All 398 children with oral swabs collected had valid Ultra results, of which 6 were positive (diagnostic yield, 2%). No drug resistance was identified by swabs.

**Table 1.** Characteristics of Children in Oral Swab Analysis by Clinical Classification

	TB, No. (%) or Median (IQR)				
Characteristic	All	Confirmed	Unconfirmed	Unlikely	Unclassifiable
Overall	399	33 (8)	269 (67)	70 (18)	27 (7)
Sex					
Female	191 (48)	17 (52)	129 (48)	33 (47)	12 (44)
Male	208 (52)	16 (48)	140 (52)	37 (53)	15 (56)
Age, mo	16 (12–35)	16 (12–36)	16 (12–34)	20 (13.25–38.5)	14 (11–17)
Weight-for-length					
*z* score	−1.90 (−3.56, −0.42)	−1.87 (−3.15, −0.44)	−1.84 (−3.51, −0.39)	−1.78 (−3.60, −0.37)	−2.78 (−4.35, −1.81)
Unknown/missing data	5	0	1	2	2
Weight-for-age					
*z* score	−2.67 (−4.50, −0.99)	−3.13 (−4.11, −0.69)	−2.57 (−4.40, −0.94)	−2.17 (−4.72, −0.97)	−3.78 (−4.62, −3.07)
Unknown/missing data	1	0	0	0	1
Malnutrition status					
SAM	188 (47)	12 (36)	126 (47)	32 (46)	18 (67)
MAM	43 (11)	6 (18)	25 (9)	9 (13)	3 (11)
Not malnourished	163 (41)	14 (42)	118 (44)	27 (39)	4 (15)
Unknown/missing data	5 (1)	1 (3)	0	2 (3)	2 (7)
HIV					
Infected	67 (17)	6 (18)	52 (19)	4 (6)	5 (19)
Exposed	75 (19)	5 (15)	58 (22)	9 (13)	3 (11)
Uninfected	256 (64)	22 (67)	158 (59)	57 (81)	19 (70)
Unknown/missing data	1 (0)	0	1	0	0
Previous diagnosis of TB					
Yes	0	0	0	0	0
No	394 (99)	32 (97)	267 (99)	69 (99)	26 (96)
Unknown/missing data	5 (1)	1 (3)	2 (1)	1 (1)	1 (4)
Hospitalized at enrollment					
Yes	222 (56)	19 (58)	146 (54)	35 (50)	22 (81)
No	176 (44)	14 (42)	123 (46)	35 (50)	4 (15)
Unknown/missing data	1 (0)	0	0	0	1 (4)
Close or household contact to someone with active pulmonary TB					
Yes	115 (29)	14 (42)	101 (38)	0	0
No	265 (66)	19 (58)	156 (58)	67 (96)	23 (85)
Unknown/missing data	19 (5)	0	12 (4)	3 (4)	4 (15)
Initiated TB treatment					
TB disease treatment^a^	303 (76)	32 (97)	241 (90)	16 (23)	14 (52)
Untreated	93 (23)	1 (3)	26 (10)	53 (76)	13 (48)
TPT only	3 (1)	0	2 (1)	1 (1)	0
TST result					
Positive	73 (18)	13 (39)	60 (22)	0	0
Negative	286 (72)	17 (52)	197 (73)	63 (90)	9 (33)
TST not done	27 (7)	1 (3)	7 (3)	2 (3)	17 (63)
Invalid^b^	13 (3)	2 (6)	5 (2)	5 (7)	1 (4)
CXR					
Abnormal, suggestive of TB disease^c^	50 (13)	7 (21)	43 (16)	0	0
Abnormal, not specific for TB disease^d^	148 (37)	11 (33)	101 (38)	28 (40)	8 (30)
Normal	167 (42)	11 (33)	109 (41)	42 (60)	5 (19)
Unable to classify	28 (7)	4 (12)	15 (6)	0	9 (33)
Missing CXR	6 (2)	0	1 (0)	0	5 (19)
Ultra and/or culture result on gastric aspirate					
Positive^e^	16 (4)	16 (48)	0	0	0
Negative^f^	374 (94)	16 (48)	268 (>99)	68 (97)	22 (81)
Unknown/invalid^g^	9 (2)	1 (4)	1 (<1)	2 (3)	5 (19)
Ultra and/or culture result on NPA 1					
Positive^e^	11 (3)	11 (33)	0	0	0
Negative^f^	385 (96)	22 (67)	269 (100)	68 (97)	26 (96)
Unknown/invalid^g^	3 (1)	0	0	2 (3)	1 (4)
Ultra and/or culture result on NPA 2					
Positive^e^	13 (3)	13 (39)	0	0	0
Negative^f^	380 (95)	20 (61)	268 (>99)	70 (100)	22 (81)
Unknown/invalid^g^	6 (2)	0	1 (<1)	0	5 (19)
Ultra and/or culture result on composite of NPA1 and NPA2					
Positive^e^	19 (5)	19 (58)	0	0	0
Negative^f^	379 (95)	14 (42)	269 (100)	70 (100)	26 (96)
Unknown/invalid^g^	1 (<1)	0	0	0	1 (4)
Ultra result on stool					
Positive^e^	12 (3)	12 (36)	0	0	0
Negative^f^	364 (91)	21 (64)	258 (96)	63 (90)	22 (81)
Unknown/invalid^g^	23 (6)	0	11 (4)	7 (10)	5 (19)
Ultra result on oral swab					
Positive^e^	6 (2)	2 (6)	3 (1)	0	1 (4)
Negative^f^	392 (98)	31 (94)	266 (99)	70 (100)	25 (93)
Unknown/invalid^g^	1 (<1)	0	0	0	1 (4)

Abbreviations: CXR, chest x-ray; LJ, Löwenstein-Jensen; MAM, moderate acute malnutrition; MGIT, Mycobacterial Growth Indicator Tube 960; NPA, nasopharyngeal aspirate; SAM, severe acute malnutrition; TB, tuberculosis; TPT, tuberculosis preventive therapy; TST, tuberculin skin test; Ultra, Xpert MTB/RIF Ultra.

^a^TB disease treatment included multiple drug regimens for drug-sensitive or drug-resistant TB disease.

^b^Invalid TSTs were those where interpretation of the result was outside 46 to 74 hours.

^c^CXR features included uncomplicated and complicated lymph node disease, miliary TB, pleural effusion, parenchyma cavitation, and Ghon focus/complex [23].

^d^CXR features included alveolar opacification (consolidation) and perihilar or interstitial infiltrates, without any CXR features suggestive of TB disease [23].

^e^At least 1 valid positive result on Ultra, MGIT, or LJ.

^f^At least 1 valid negative result on Ultra, MGIT, or LJ and no valid positive result.

^g^No available and valid result on Ultra, MGIT, and LJ. Unknown/invalid results include missing results (from samples not collected) and unknown/invalid results (including cultures contaminated or identification unknown; Ultra error, no result, or invalid).

### Diagnostic Accuracy of Oral Swabs

Sensitivity of double swabs was low per the MRS (6.9%; 95% CI, 1.9%–22.0%) and CRS (1.8%; 95% CI, .8%–4.1%). Results were similar for single and double swabs combined (Table 2). In subgroup analyses (Supplementary Table 2), the highest sensitivity occurred among CWH for diagnosing confirmed TB (16.7%; 95% CI, 3.0%–56.4%). Comparison of swab-processing methods suggested a trend for higher sensitivity with Cepheid SR per the MRS. However, we did not power the study for subgroup comparisons, subgroups were small, and 95% CIs overlapped. Specificity was consistently high for the whole cohort (>99%) and subgroups (>97%).

**Table 2.** Accuracy of Xpert MTB/RIF Ultra Assay on Oral Swabs for Diagnosing Tuberculosis According to Different Reference Standards

Swabs: Reference Standard	No. of Participants^a^	n/N^b^	Sensitivity (95% CI), %	n/N^b^	Specificity (95% CI), %
**Double only**					
Strict	93	2/29	6.9 (1.9–22.0)	64/64	100 (94.3–100.0)
Microbiological	344	2/29	6.9 (1.9–22.0)	312/315	99.0 (97.2–99.7)
Clinical	345	5/281	1.8 (.8–4.1)	64/64	100 (94.3–100.0)
**Single and double**					
Strict	103	2/33	6.1 (1.7–19.6)	70/70	100 (94.8–100.0)
Microbiological	371	2/33	6.1 (1.7–19.6)	335/338	99.1 (97.4–99.8)
Clinical	372	5/302	1.7 (.7–3.8)	70/70	100 (94.8–100.0)

^a^Including only participants with valid swab Xpert MTB/RIF Ultra results and clinical classifications.

^b^Number of positive or negative oral swab results (n) / total number classified as reference standard positive or negative (N).

Supplementary Table 3 summarizes the clinical, immunologic, and radiologic features of the 6 children with a positive oral swab Ultra result. Two children (33%) had confirmed TB, 3 (50%) had unconfirmed TB, and 1 (17%) was unclassifiable due to missed follow-ups. There were no significant differences in clinical features between children with positive and negative swab results (Supplementary Table 4).

**Figure 3.**
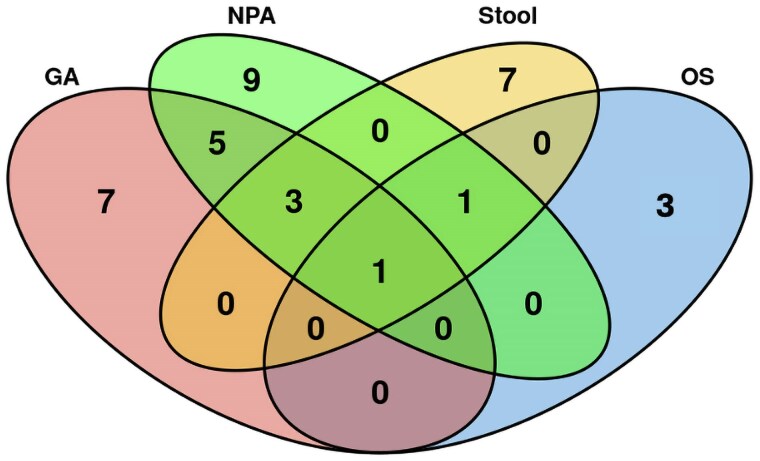
Venn diagram of the yield of Xpert MTB/RIF Ultra and culture on each reference test and Xpert MTB/RIF Ultra on oral swabs in children testing positive per the clinical reference standard (n = 302). Abbreviations: GA, gastric aspirate; NPA, nasopharyngeal aspirate (composite of NPA 1 and NPA 2); OS, oral swab.

Figure 3 shows the yield of each reference test and the added yield of oral swab Ultra in children positive per the CRS. Oral swab testing increased the percentage of total TB cases with microbiological confirmation from 11% (33/302) to 12% (36/302). Ultra testing on oral swab and stool samples alone detected 42% (15/36) of cases, identifying 10 additional microbiologically confirmed cases as compared with the combination of Ultra and culture on 2 NPAs and 1 GA.

Supplementary Table 5 shows the Ultra semiquantitative results on each respiratory sample type vs the oral swab specimen. Generally, the respiratory specimens had results that indicated the same or higher bacillary load as the oral swabs. However, in 4 participants where *Mtb* was not detected by all reference standard tests, the oral swab yielded a trace result.

## DISCUSSION

We present the first data using Ultra on double oral swabs collected at 1 time point for pulmonary TB diagnosis in children aged <5 years, including swabs processed by an SR-free heat method. We were able to collect swabs from nearly all children. Despite concerns of cartridge overpressurization errors from heat-based processing methods [24], no error or invalid Ultra results were seen, suggesting that double-swab collection with Ultra testing is highly feasible. In keeping with other pediatric studies [9, 11, 14], specificity was very high (>99%), with no positive swabs in the unlikely TB group, supporting that a positive swab Ultra result can rule in TB. However, the low sensitivity of Ultra on double swabs in our study (7%) suggests that multiple swabs cannot currently replace traditional, more invasive reference specimens in young children.

Our double-swab sensitivity of 7% with an MRS or 2% with a CRS is lower than the other published pediatric study of Ultra by Cox et al, who recruited children aged ≤15 years in South Africa. In children aged <5 years, they noted a sensitivity of 22% with an MRS and 9% with a CRS and a sensitivity of 18% with an MRS [11]. However, the number of children aged <5 years (n = 81) was fewer than ours (n = 344). They also had a higher proportion of hospitalized children with TB, who are more likely to have severe disease, which could contribute to the higher sensitivity of swabs in their study. Differences in processing, swab type, oral sampling location, and testing of frozen swabs could also account for differences. Other pediatric studies evaluating in-house polymerase chain reaction (PCR) assays showed a sensitivity of 8% to 42% with an MRS [9, 14] and 5% to 42% with a CRS [9, 14, 17]. Yet, differences included testing of a single swab [14, 17], swab type [9, 14, 17], freezing of swabs [9, 14], and an older cohort with a higher bacillary burden [9, 14], all of which may influence sensitivity and limit comparison.

In subgroup analyses, swab sensitivity was highest among CWH. This trend was noted in other pediatric studies of oral swabs [11], stool [25], and urine [26] and may be due to more severe TB disease and more advanced pathology in CWH. Determine TB LAM Ag (Abbott Laboratories) testing on urine and Ultra on stool had MRS sensitivities of 47% [26] and 88% [27] in CWH, respectively, suggesting that urine and stool are more likely to detect TB than oral swabs in this subgroup.

Swabs in our study were processed by either SR or heat inactivation. Data on the optimal processing method are mixed [16]. Chilambi et al showed that processing spiked adult tongue swabs with different concentrations of SR had a lower *Mtb* limit of detection as compared with the SR-free heat inactivation protocol [28], whereas Andama et al found that heat processing of a single spiked tongue swab yielded a lower limit of detection when compared with swabs processed with SR [12]. Our subgroup analysis on processing methods must be interpreted with caution due to overlapping 95% CIs and limited power due to the small number of confirmed cases. Rather than a head-to-head comparison, the processing method depended on when the child was recruited, and temporal differences in factors that influence sensitivity may play some role in observed differences. Clinical studies with direct comparisons on the same participants are needed to identify the optimal processing strategy in young children.

Swabs detected *Mtb* in 4 symptomatic children with negative microbiological reference test results. Excluding the child lost to follow-up, the clinical information of these cases suggested that they were true disease positives rather than instances of bacillus detection from previously treated TB or latent infection. While the absolute number of children in whom TB was identified through oral swabs alone was small, it increased the total number of confirmed TB cases by 1% and might be clinically relevant. Previous pediatric studies reported an increase in yield of microbiologically diagnosed TB cases with swabs, although the proportion of confirmed cases varied from 1% [11] to 13% [14] and 48% [9]. Additionally, the combination of stool and oral swab Ultra yielded 10 additional positive results over GA/NPA (Figure 3). Oral swabs could therefore still play a complementary role in existing diagnostic algorithms for TB to increase microbiological yield where Ultra testing is already established, especially since diagnosis improves when a combination of different samples is tested [29, 30]. Studies assessing the diagnostic accuracy and feasibility of collecting minimally invasive sample combinations are needed to determine optimal sampling strategies for children aged <5 years.

The ease of swab collection could compensate to some extent for the limited sensitivity, as compared with less feasible specimens such as GA, NPA, or induced sputum [31]. However, improvement of sensitivity above what we observed in our study is first needed. Ultra was developed for analyzing sputum samples, so it may always be limited in detecting *Mtb* from oral swabs [12]. Olson et al applied 2 swab-specific protocols with dual-target quantitative PCR to archived swabs from adults in South Africa with presumptive TB, involving the use of foam swabs and sedimentation to increase collected *Mtb* bacillus biomass and the use of hybridization probes immobilized on magnetic beads to selectively concentrate *Mtb* DNA [32]. Both methods were more sensitive for TB than evaluating the same participants with Ultra and Molbio Truenat MTB Ultima, with high specificity maintained [8]. Similarly, Steadman et al demonstrated that a novel laboratory-based protocol involving heat, high-energy bead beating, and a high-volume quantitative PCR assay had 93% sensitivity and 99% specificity per an MRS in symptomatic adults in Uganda [33]. Although manual laboratory-based PCR assays can have false positives [9] and be challenging to automate for point-of-care testing [32], the expansion of near-patient swab-based molecular platforms developed for COVID-19 testing suggests that such technical innovations are possible and could be adaptable for TB [34, 35]. Yet, many novel processing methods and manual in-house PCR tests in adults performed better in those with higher semiquantitative Ultra sputum results [8, 32, 33, 36], suggesting that they may be inadequate in paucibacillary populations such as young children. A consensus statement on pediatric TB diagnostic development recommended that pathogen-based tests be evaluated in children in parallel to adults when sample collection has minimal risk [37]. Prospective studies evaluating novel swab protocols designed to improve sensitivity need to be promptly performed in young children to bridge this gap.

Our study has limitations. Although recruitment occurred across outpatient and inpatient settings at 2 sites in Uganda, the single-country design limits generalizability. Our study focus was the use of double swabs, although a small number of single swabs (30/398, 8%) were collected at the beginning of the study, which could collect less mycobacterial biomass than double swabs [9, 12]. However, analyses comparing single and double swabs combined and double swabs alone were similar (Table 2).

We acknowledge variations in swab collection, processing, and testing methods, which may have influenced our results by limiting the sample size per group. Nonetheless, these modifications were guided by emerging laboratory findings to improve sensitivity and address operational and implementation challenges. We did not compare different locations within the oral cavity. Swabbing location has been noted to influence diagnostic yield in older children and adults, with differing results [10, 11]. However, our protocol of swabbing the whole mouth is more similar to “real life” conditions, where it can be difficult to target distinct locations in the mouth of a poorly cooperative infant. Finally, the number of confirmed cases with a positive culture result was too small to allow a comparison between culture and Ultra from any respiratory specimen. Imperfect pediatric TB reference standards make interpretation of swab performance difficult. The large unconfirmed TB group in our study may be partly explained by (1) the low threshold for starting TB treatment in symptomatic children who were <2 years old or had HIV and (2) the fact that some children with non-TB diseases will appear to respond to TB treatment, since symptoms naturally resolve and TB treatment includes broad-spectrum antibiotics. If children without TB are misclassified as having unconfirmed TB, sensitivity will be underestimated, whereas misclassification of children with TB as unlikely TB will underestimate specificity. Despite these limitations, this is the first evaluation of Ultra on double swabs on children aged <5 years and on heat inactivation as a processing method in children. Unlike previous pediatric studies [9, 11, 14], most swabs were tested prospectively, as would be done in clinical practice. Our extensive microbiological diagnostic evaluation, strict eligibility criteria, and follow-up over 6 months for assessing treatment response will have reduced misclassification of children. Our large cohort of young children with a high representation of CWH and malnutrition also represents the most difficult-to-diagnose populations.

In summary, our study suggests that the low sensitivity of Ultra on double oral swabs with either SR or heat inactivation precludes its role as a principal diagnostic approach for TB in children aged <5 years. However, the high rates of swab collection and valid results—in addition to the fact that swabs detected TB in a small but important group that otherwise would not have been microbiologically confirmed—suggest that swabs should not be disregarded as a useful additional sample for pediatric TB. A priority for TB research is to increase swab diagnostic sensitivity in paucibacillary populations. Recent results for adults that move away from Ultra and maximize the pickup of *Mtb* or increase lysis efficiency are promising [32, 33], but performance in children remains unknown. Prospective diagnostic evaluations of swab-specific protocols with head-to-head comparisons need to be performed in young children early in the diagnostic research pipeline.

## Supplementary Material

ofaf206_Supplementary_Data

## Supplementary Data

Supplementary materials are available at *Open Forum Infectious Diseases* online. Consisting of data provided by the authors to benefit the reader, the posted materials are not copyedited and are the sole responsibility of the authors, so questions or comments should be addressed to the corresponding author.

## Notes

***Acknowledgments.*** We thank all participants and their parents/guardians for their participation in the study. *NOD-pedFEND consortium authors:* Gerald Agaba Muzorah, Gideon Ahimbisibwe, Sharley Aloyo, Sheillah Ansiima, Kiranjot Arora, Henry Balwa, Kisegerwa Bashir, April Borkman, Eric Bugumirwa, George Haumba, David Hom, Florence Kalawa, Florence Kalembe, Samuel Kasibante, Nakitto Aisha Kawwoya, Daisy Kyamulabi, Rose Magala, Frank Matovu, Angella Mirembe, Benedicto Mugabi, Nelson Muhumuza, Levi Mwanga, Mary Nakagwa, Brenda Sharon Nakalanda, Lydia Nakiyingi, Aminah Nakyazze, Rose Namaganda, Hellen Nassolo, Claire Night, Gloria Ninsiima, Israel Odongo, Kamulegeya Rogers, Willy Ssengooba, Jessica Tagobera, Abner Tagoola, Ann Tufariello, Agnes Turyamubona.

***Author contributions*.** Conceptualization: N. K., F. W. B., N. M., E. M. B., R. S. Methodology: N. K., F. W. B., N. M., E. M. B., R. S., M. d. V., A. P.-N., D. Armstrong, M. R. Writing–original draft: N. K. and K. A. M. Writing–review and editing: all authors. Project administration: N. K., F. W. B., N. M., E. D., E. N., G. N. Investigation: K. A. M., E. N., S. R. B., S. N., G. N., F. K. L., M. K., A. N., J. P. L., R. N., P. M., G. P. K., P. M., H. S. S., M. P., E. W. Data management and curation: R. U., R. D., R. P. Formal analysis: N. K., N. M., R. D., R. P., S. K. Supervision: E. M. B., S. K., G. P. K., R. S. Funding acquisition: D. Alland, S. E. D., J. E., M. J., M. R., A. R. K., R. S.

***Financial support*.** This work was supported by grants from the National Institute of Allergy and Infectious Diseases of the National Institutes of Health (AI152159, AI152084); a fellowship from the European Society for Paediatric Infectious Diseases (to E. M. B.); and the Marshall Aid Commemoration Commission (to N. M.).

***Potential conflicts of interest*.** D. Alland holds patents related to tuberculosis detection and drug treatment. He also receives royalty payments for 1 or more of these patents that have been licensed by Rutgers University to Cepheid. All other authors report no potential conflicts.
