# Supplementary material for: Oral Swab Testing With Xpert MTB/RIF Ultra for the Diagnosis of Tuberculosis in Children Aged <5 Years in Uganda: An Exploratory Interim Analysis of Diagnostic Accuracy in the NOD-pedFEND Cohort
Source: Open Forum Infect Dis. 2025 Apr 8;12(4):ofaf206. doi: 10.1093/ofid/ofaf206 (PMC12013808; doi:10.1093/ofid/ofaf206)
Supplement: ofaf206_Supplementary_Data [file ofaf206_supplementary_data.docx]

**Supplementary material for oral swab paper**

**Summary**

Supplementary Figure 1: Clinical classification definitions and reference standards for diagnostic accuracy of swabs

Supplementary Table 1: Changes to oral swab collection, processing and timing of Ultra testing during study period

Supplementary Table 2: Accuracy of Ultra assay on oral swabs for diagnosing TB according to different reference standards- subgroup analyses

Supplementary Table 3: Details of clinical, immunological and radiological information for six children with positive oral swab Ultra results

Supplementary Table 4: Comparison of children with positive and negative Ultra oral swab results

Supplementary Table 5: Ultra semi-quantitative levels in the oral swab vs. respiratory reference samples

*
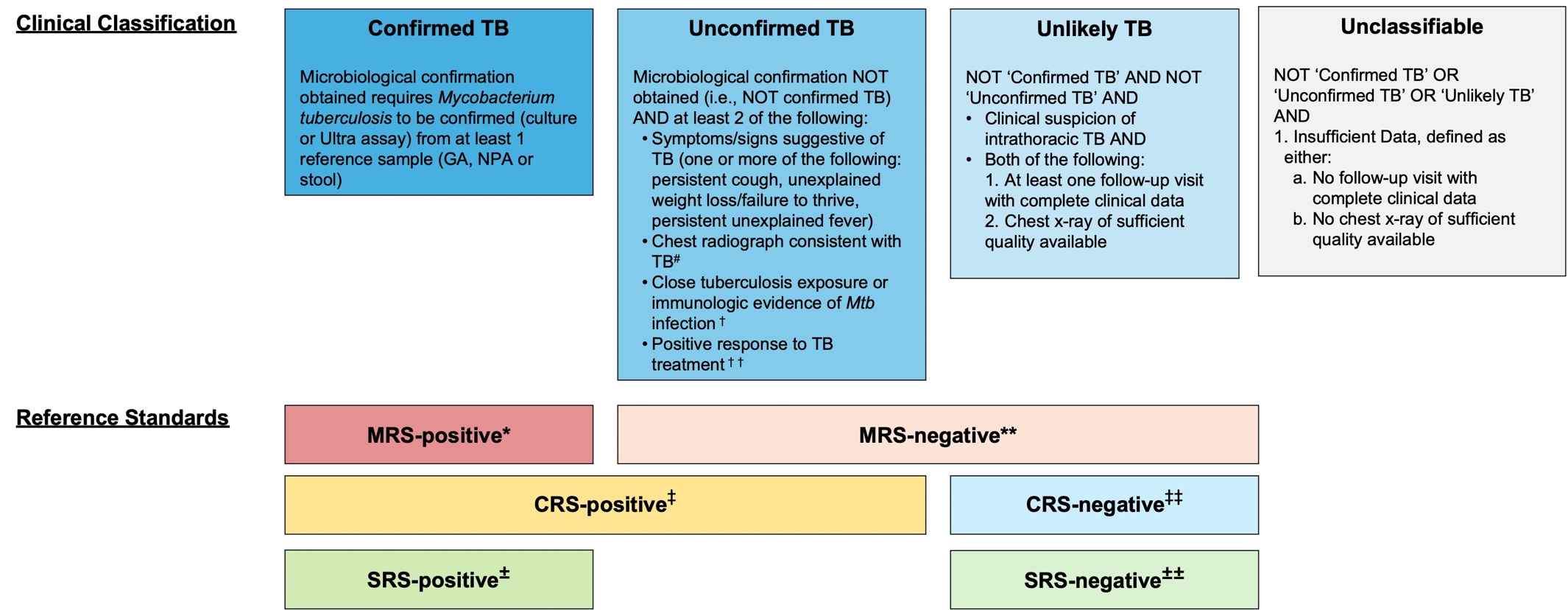
*

***Supplementary Figure 1: Clinical classification definitions and reference standards for diagnostic accuracy of swabs***

** Participants MRS-positive meet the Clinical Case Definition of Confirmed TB*

*** Participants MRS-negative meet the Clinical Case Definition of either Unconfirmed TB or Unlikely TB AND must have 6 negative tests for MTB.*

*‡ Participants CRS-positive meet either Clinical Case Definitions of Confirmed TB or Unconfirmed TB.*

*‡‡ Participants CRS-negative meet the Clinical Case Definition of Unlikely TB.*

*± Participants SRS-positive meet the Clinical Case Definition of Confirmed TB (identical to the MRS-positive).*

*±± Participants SRS-negative meet the Clinical Case Definition of Unlikely TB AND must have 6 negative tests for MTB.*

*#. Digital chest x-rays were assessed for features suggestive of TB disease by an independent and blinded three-person expert panel.*

*† Immunological evidence of Mtb infection was defined by a positive TST.*

*†† Positive response to TB treatment defined by 1) opinion of attending clinician at end of treatment and 2) resolution of all TB symptoms at 2 months post-treatment and/or end of treatment.*

*SRS = Strict Reference Standard, MRS = Microbiological Reference Standard, CRS = Composite Reference Standard, GA = gastric aspirate, NPA = nasopharyngeal aspirate, MTB = Mycobacterium tuberculosis*

**Supplementary Table 1: Changes to oral swab collection, processing and timing of Ultra testing during study period**

|  | **Aug 2021-Dec 2021** | **Jan 2022 to July 2022** | **Aug 2022 to Jun 2023** | **July 2023 to Feb 2024** |
| --- | --- | --- | --- | --- |
| **Number of oral swabs** | Single | Double | Double | Double |
| **Processing method** | Cepheid SR with 0.9ml PBS | Cepheid SR with 0.9ml PBS | Cepheid SR with 0.9ml PBS | Heat inactivation with 0.9ml TE |
| **Timing of Ultra testing** | Retrospective | Retrospective | Prospective | Prospective |
| **No. (%) of children** | 30 (7.5%) | 79 (19.8%) | 168 (42.2%) | 121 (30.4%) |

Most children had double oral swabs collected (two consecutive swabs stored in a single cryovial at one timepoint) which were tested with Ultra prospectively. Only children with oral swabs collected and complete NIH classifications are included here (total N = 398)

TE= Tris EDTA, PBS = Phosphate-buffered saline, SR = Sample reagent

***Supplementary Table 2: Accuracy of Ultra assay on oral swabs for diagnosing TB according to different reference standards- subgroup analyses***

|  | **Strict reference standard** | | | | **Microbiological reference standard** | | | | **Composite reference standard** | | | |
| --- | --- | --- | --- | --- | --- | --- | --- | --- | --- | --- | --- | --- |
| **Subgroup** | **n/N** | **Sensitivity (95% CI), %** | **n/N** | **Specificity (95% CI), %** | **n/N** | **Sensitivity (95% CI), %** | **n/N** | **Specificity (95% CI), %** | **n/N** | **Sensitivity (95% CI), %** | **n/N** | **Specificity**  **(95% CI), %** |
| With HIV infection | 1/6 | 16.7  (3.0, 56.4) | 4/4 | 100  (51.0, 100.0) | 1/6 | 16.7  (3.0, 56.4) | 55/55 | 100  (93.5, 100.0) | 1/58 | 1.7  (0.3, 9.1) | 4/4 | 100  (51.0, 100.0) |
| Without HIV infection | 1/27 | 3.7  (0.7, 18.3) | 66/66 | 100  (94.5, 100.0) | 1/27 | 3.7  (0.7, 18.3) | 279/282 | 98.9  (96.9, 99.6) | 4/243 | 1.6  (0.6, 4.2) | 66/66 | 100  (94.5, 100.0) |
| Age <12 months | 1/7 | 14.3  (2.6, 51.3) | 14/14 | 100  (78.5, 100.0) | 1/7 | 14.3  (2.6, 51.3) | 73/75 | 97.3  (90.8, 99.3) | 3/68 | 4.4  (1.5, 12.2) | 14/14 | 100  (78.5, 100.0) |
| Age≥12 months | 1/26 | 3.8  (0.7, 18.9) | 56/56 | 100  (93.6, 100.0) | 1/26 | 3.8  (0.7, 18.9) | 262/263 | 99.6  (97.9, 99.9) | 2/234 | 0.9  (0.2, 3.1) | 56/56 | 100  (93.6, 100.0) |
| Acute malnutrition^+^ | 2/18 | 11.1  (3.1, 32.8) | 41/41 | 100  (91.4, 100.0) | 2/18 | 11.1  (3.1, 32.8) | 191/191 | 100  (98.0, 100.0) | 2/169 | 1.2  (0.3, 4.2) | 41/41 | 100  (91.4, 100.0) |
| No malnutrition | 0/14 | 0  (0.0, 21.5) | 27/27 | 100  (87.5, 100.0) | 0/14 | 0  (0.0, 21.5) | 142/145 | 97.9  (94.1, 99.3) | 3/132 | 2.3  (0.8, 6.5) | 27/27 | 100  (87.5, 100.0) |
| Cepheid SR/PBS  (all swabs)^#^ | 2/26 | 7.7  (2.1, 24.1) | 48/48 | 100  (92.6, 100.0) | 2/26 | 7.7  (2.1, 24.1) | 231/231 | 100  (98.4, 100.0) | 2/210 | 1.0  (0.3, 3.4) | 48/48 | 100  (92.6, 100.0) |
| Heat inactivation/TE  (all swabs)^#^ | 0/7 | 0  (0.0, 35.4) | 22/22 | 100  (85.1, 100.0) | 0/7 | 0  (0.0, 35.4) | 104/107 | 97.2  (92.1, 99.0) | 3/92 | 3.3  (1.1, 9.2) | 22/22 | 100  (85.1, 100.0) |
| Cepheid SR/PBS  (double swabs only) | 2/22 | 9.1  (2.5, 27.8) | 42/42 | 100  (91.6, 100.0) | 2/22 | 9.1  (2.5, 27.8) | 208/208 | 100  (98.2-100.0) | 2/189 | 1.1  (0.3, 3.8) | 42/42 | 100  (91.6 100.0) |
| Heat inactivation/TE (double swabs only) | 0/7 | 0  (0.0, 35.4) | 22/22 | 100  (85.1, 100.0) | 0/7 | 0  (0.0, 35.4) | 104/107 | 97.2  (92.1, 99.0) | 3/92 | 3.3  (1.1, 9.2) | 22/22 | 100  (85.1, 100.0) |

*Only including participants with valid Ultra results and clinical classifications*

*n= Number of positive or negative swab results.*

*N = Total number classified as reference standard positive or negative*

*^*^ Includes both severe and moderate acute malnutrition*

*^#^Includes both double and single swabs combined.*

*CI = confidence interval, TE= Tris EDTA, PBS = phosphate-buffered saline, SR = sample reagent*

**Supplementary Table 3: *Details of clinical, immunological and radiological information for six children with positive oral swab Ultra results***

| **Age in**  **months** | **HIV status** | **Previous history of TB** | **Previous medication in last 3 months** | **Presenting symptoms** | **Physical findings** | **WfH Z-score at baseline** | **SAM** | **TB close contact person** | **Malaria test result** | **TST result (induration, mm)** | **Active TB treatment started** | **Positive response to treatment*** | **CXR**  **assessment**** | **Reference microbiological results (NPA, GA, Stool) and SQ result** | **Swab collection and processing** | **Swab Ultra semiquantitative result** | **Clinical classification** |
| --- | --- | --- | --- | --- | --- | --- | --- | --- | --- | --- | --- | --- | --- | --- | --- | --- | --- |
| **5** | Positive | **No** | Antibiotics, antifungals | Cough, difficulty in breathing, fever, fatigue, weight loss, and night sweats | Cervical lymphadenopathy, tachypnoea, reduced air entry, crepitations, hepatomegaly | -5.05 | Yes | Family friend clinically diagnosed with pulmonary TB | Negative | Negative  (0mm) | Yes, at baseline | Yes | Perihilar and interstitial infiltrates and alveolar opacification: CXR abnormal, with no specific features for TB. | NPA1- Ultra positive (low), MGIT positive  NPA2- Ultra positive (medium), MGIT positive  GA- not collected  Stool- Ultra positive (very low) | Double swab, in PBS, Cepheid SR, Ultra prospectively tested | Low | Confirmed TB |
| **36** | Negative | **No** | Antibiotics, nutritional therapy | Cough, difficulty in breathing, fever, fatigue, weight loss, and night sweats | Cervical lymphadenopathy | -2.71 | No | None reported | Negative | Negative  (0mm) | Yes, at baseline | Yes | Miliary pattern: features specific for TB | NPA1- Ultra positive (medium) with RIF resistance, MGIT & LJ positive  NPA2- Ultra positive (low), MGIT & LJ positive  GA- Ultra positive (medium), MGIT & LJ positive,  Stool- Ultra positive (low) | Double swab, in PBS, Cepheid SR, Ultra prospectively tested | Very low | Confirmed TB |
| 8 | Negative | No | Antibiotics, antimalarials | Cough, difficulty in breathing, fever and night sweats | Signs of respiratory distress: nasal flaring and recession | 1.07 | No | None reported | Negative | Negative (2mm) | Yes, at baseline | Yes | Normal | All samples negative | Double swab, in TE buffer, heat inactivation, Ultra prospectively tested | Trace | Unconfirmed TB |
| 16 | Negative | No | Antibiotics | Cough, fever, fatigue and weight loss | None | -0.61 | No | None reported | Negative | Not done | Yes, at baseline | Unable to assess-caregivers moved away from study site and withdrew from the study | Perihilar infiltrates and alveolar opacification: CXR abnormal, with no specific features for TB | All samples negative | Double swab, in TE buffer, heat inactivation, Ultra prospectively tested | Trace | Unclassifiable |
| 8 | Negative | No | Antibiotics, antimalarials, nutritional therapy,  vitamin K, tranexamic acid | Fever, fatigue, weight loss, jaundice and oedema | None | -1.79 | No | Mother clinically diagnosed with pulmonary TB | Negative | Positive (17mm) | Yes, at 2 weeks into the study | Yes | Interstitial infiltrates:  CXR abnormal, with no specific features for TB | All samples negative | Double swab, in TE buffer, heat inactivation, Ultra prospectively tested | Trace | Unconfirmed TB |
| 13 | Negative | No | Antibiotics | Cough, fever, fatigue and weight loss | Cervical and axillary lymphadenopathy, reduced air entry and crepitations | 1.03 | No | Family member in household microbiologically diagnosed with pulmonary TB | Negative | Negative (3mm) | Yes, at baseline | Yes | Normal | All samples negative | Double swab, in TE buffer, heat inactivation, Ultra prospectively tested | Trace | Unconfirmed TB |

** Positive response to TB treatment defined by 1) opinion of attending clinician at end of treatment and 2) resolution of all TB symptoms and weight gain if malnourished at 2 months post-treatment and/or end of treatment.*

*** As assessed by an independent three-person panel, blinded to TB reference tests and clinical information*

*WfH = weight for height/length, SAM = severe acute malnutrition, TST = tuberculin skin test, CXR = chest-x-ray, SQ = semiquantitative*

**Supplementary Table 4: Comparison of children with positive and negative Ultra oral swab results**

| **Characteristic** |  | **Oral swab Ultra negative (N=392)** | **Oral swab Ultra positive (N=6)** | **P-Value** |
| --- | --- | --- | --- | --- |
| **Sex** | Female | 186 (47%) | 4 (67%) | 0.431 |
|  | Male | 206 (53%) | 2 (33%) |  |
| **Age** | Median age in months, (IQR) | 17.0 (12.0, 35.5) | 10.5 (8.0, 16.0) | 0.093 |
| **Weight-for-length** | Median weight-for-length z score (IQR) | -1.9 (-3.6, -0.4) | -1.2 (-2.7, 1.0) | 0.407 |
| **Weight-for-age** | Median weight-for-age z score, (IQR) | -2.7 (-4.5, -1.0) | -2.2 (-4.3, -0.4) | 0.669 |
| **Malnutrition Status** | SAM | 187 (48%) | 1 (17%) | 0.295 |
|  | MAM | 42 (11%) | 1 (17%) |  |
|  | Not malnourished | 159 (41%) | 4 (67%) |  |
|  | Missing or incomplete data | 4(1%) | 0 |  |
| **HIV** | HIV infected | 65 (17%) | 1 (17%) | 1.000 |
|  | HIV negative | 326 (83%) | 5 (83%) |  |
|  | Unknown | 1 (0%) | 0 (0%) |  |
| **Previous diagnosis of TB** | No | 388 (99%) | 6 (100%) | 1.000 |
|  | Unknown | 4 (1%) | 0 (0%) |  |
| **Hospitalized at enrollment** | Yes | 219 (56%) | 3 (50%) | 1.000 |
|  | No | 173 (44%) | 3 (50%) |  |
| **Close or household contact to someone with active pulmonary TB** | Yes | 113 (29%) | 2 (33%) | 1.000 |
|  | No | 261 (67%) | 4 (67%) |  |
|  | Unknown | 18 (5%) | 0 (0%) |  |
| **Initiated TB treatment** | TB disease treatment*^*^* | 297 (76%) | 5 (83%) | 1.000 |
|  | Untreated | 92 (23%) | 1 (17%) |  |
|  | IPT/TPT only | 3 (1%) | 0 (0%) |  |
| **TST result** | Positive TST | 72 (18%) | 1 (17%) | 0.518 |
|  | Negative TST | 282 (72%) | 4 (67%) |  |
|  | Invalid** | 13 (3%) | 0 (0%) |  |
|  | TST not done | 25 (6%) | 1 (17%) |  |
| **Chest x-ray** | Abnormal, suggestive of TB disease*** | 49 (13%) | 1 (17%) | 0.834 |
|  | Abnormal, not specific for TB disease**** | 145 (37%) | 3 (50%) |  |
|  | Normal | 165 (43%) | 2 (33%) |  |
|  | Unable to classify | 28 (7%) | 0 (0%) |  |
|  | Missing CXR | 5 (1%) | 0 |  |

*Continuous variables were compared using the Wilcoxon Test and categorical variables were compared using the Fishers’ exact test. P-values were two-sided.*

** TB disease treatment included multiple drug regimens for drug-sensitive or drug-resistant TB disease*

***Invalid TST were those where interpretation of the result was outside 46-74 hours*

**** CXR features included uncomplicated and complicated lymph node disease, miliary TB, pleural effusion, parenchyma cavitation and Ghon focus/complex^23^*

*****CXR features included alveolar opacification (consolidation) and perihilar or interstitial infiltrates, without any CXR features suggestive of TB disease^23^*

*SAM = Severe acute malnutrition , MAM = Moderate acute malnutrition , TST= Tuberculin skin test, TPT: TB preventative therapy, CXR: Chest x-ray*

**Supplementary Table 5: Ultra semi-quantitative levels in the oral swab vs. respiratory reference samples***

|  | | **Oral swab** | | | | | | |  |
| --- | --- | --- | --- | --- | --- | --- | --- | --- | --- |
| **Respiratory Specimen** |  | **Negative (N=392)** | **Trace (N=4)** | **Very Low (N=1)** | **Low (N=1)** | **Medium**  **(N=0)** | **High**  **(N=0)** | **Unknown (N=1)** | **Total (N=399)** |
| Gastric aspirate | Negative | **372 (99%)** | 4 (1%) | 0 | 0 | 0 | 0 | 0 | 376 |
|  | Trace | 9 (100%) | **0** | 0 | 0 | 0 | 0 | 0 | 9 |
|  | Very Low | 3 (100%) | 0 | **0** | 0 | 0 | 0 | 0 | 3 |
|  | Low | 1 (100%) | 0 | 0 | **0** | 0 | 0 | 0 | 1 |
|  | Medium | 0 | 0 | 1 (100%) | 0 | **0** | 0 | 0 | 1 |
|  | High | 0 | 0 | 0 | 0 | 0 | **0** | 0 | 0 |
|  | Missing data | 7 | 0 | 0 | 1 | 0 | 0 | 1 | 9 |
| Nasopharyngeal aspirate 1 | Negative | **383 (99%)** | 4 (1%) | 0 | 0 | 0 | 0 | 0 | 387 |
|  | Trace | 4 (100%) | **0** | 0 | 0 | 0 | 0 | 0 | 4 |
|  | Very Low | 1 (100%) | 0 | **0** | 0 | 0 | 0 | 0 | 1 |
|  | Low | 1 (50%) | 0 | 0 | **1 (50%)** | 0 | 0 | 0 | 2 |
|  | Medium | 1 (50%) | 0 | 1 (50%) | 0 | **0** | 0 | 0 | 2 |
|  | High | 0 | 0 | 0 | 0 | 0 | **0** | 0 | 0 |
|  | Missing data | 2 | 0 | 0 | 0 | 0 | 0 | 1 | 3 |
| Nasopharyngeal aspirate 2 | Negative | **377 (99%)** | 4 (1%) | 0 | 0 | 0 | 0 | 0 | 381 |
|  | Trace | 6 (100%) | **0** | 0 | 0 | 0 | 0 | 0 | 6 |
|  | Very Low | 3 (100%) | 0 | **0** | 0 | 0 | 0 | 0 | 3 |
|  | Low | 1 (50%) | 0 | 1 (50%) | **0** | 0 | 0 | 0 | 2 |
|  | Medium | 0 | 0 | 0 | 1 (100%) | **0** | 0 | 0 | 1 |
|  | High | 0 | 0 | 0 | 0 | 0 | **0** | 0 | 0 |
|  | Missing data | 5 | 0 | 0 | 0 | 0 | 0 | 1 | 6 |
| Nasopharyngeal aspirate (composite of 2 specimens) | Negative | **376 (99%)** | 4 (1%) | 0 | 0 | 0 | 0 | 0 | 380 |
|  | Trace | 9 (100%) | **0** | 0 | 0 | 0 | 0 | 0 | 9 |
|  | Very Low | 4 (100%) | 0 | **0** | 0 | 0 | 0 | 0 | 4 |
|  | Low | 2 (100%) | 0 | 0 | **0** | 0 | 0 | 0 | 2 |
|  | Medium | 1 (33%) | 0 | 1 (33%) | 1 (33%) | **0** | 0 | 0 | 3 |
|  | High | 0 | 0 | 0 | 0 | 0 | **0** | 0 | 0 |
| Stool | Negative | **361 (99%)** | 3 (1%) | 0 | 0 | 0 | 0 | 0 | 364 |
|  | Trace | 9 (100%) | **0** | 0 | 0 | 0 | 0 | 0 | 9 |
|  | Very Low | 0 | 0 | **0** | 1 (100%) | 0 | 0 | 0 | 1 |
|  | Low | 1 (50%) | 0 | 1 (50%) | **0** | 0 | 0 | 0 | 2 |
|  | Medium | 0 | 0 | 0 | 0 | **0** | 0 | 0 | 0 |
|  | High | 0 | 0 | 0 | 0 | 0 | **0** | 0 | 0 |
|  | Missing data | 21 | 1 | 0 | 0 | 0 | 0 | 1 | 23 |
| Composite of all specimens used for the reference standard | Negative | **363 (99%)** | 4 (1%) | 0 | 0 | 0 | 0 | 0 | 367 |
|  | Trace | 19 (100%) | **0** | 0 | 0 | 0 | 0 | 0 | 19 |
|  | Very Low | 5 (100%) | 0 | **0** | 0 | 0 | 0 | 0 | 5 |
|  | Low | 4 (100%) | 0 | 0 | **0** | 0 | 0 | 0 | 4 |
|  | Medium | 1 (33%) | 0 | 1 (33%) | 1 (33%) | **0** | 0 | 0 | 3 |
|  | High | 0 | 0 | 0 | 0 | 0 | **0** | 0 | 0 |
|  | Missing data | 0 | 0 | 0 | 0 | 0 | 0 | 1 | 1 |

*** Semi-quantitative results for *Mtb* are negative if not detected and if detected, the semi-quantitative level result is shown. Cell percentages are row percentages. Bolded cells have concordant results on the two specimen types. Unknown Ultra result on oral swab is from the child who was withdrawn before sample collection.
